# Horn fly transcriptome data of ten populations from the southern United States with varying degrees and molecular mechanisms of pesticide resistance

**DOI:** 10.1016/j.dib.2023.109272

**Published:** 2023-05-30

**Authors:** Kylie G. Bendele, Felix D. Guerrero, Kimberly H. Lohmeyer, Lane D. Foil, Richard P. Metz, Charles D. Johnson

**Affiliations:** a USDA-ARS Knipling-Bushland US Livestock Insects Research Laboratory, 2700 Fredericksburg Rd., Kerrville, TX 78028, USA; b Massey University, School of Veterinary Science, Genetics and Molecular Biology, Private Bag 11 222, Palmerston North, Manawatu-Wanganui 4442, New Zealand; c Department of Entomology, Louisiana State University Agriculture Center, Baton Rouge, LA 70803, USA; d Genomics and Bioinformatics Service, Texas A&M AgriLife Research, 1500 Research Parkway, Room 250, Centeq Building A, College Station, TX 77845, USA

**Keywords:** *Haematobia irritans*, Horn fly, Illumina sequencing, Insecticide resistance, Transcriptome, Pesticide resistance

## Abstract

*Haematobia irritans irritans* (Linnaeus, 1758: Diptera: Muscidae), the horn fly, is an external parasite of penned and pastured livestock that causes a major economic impact on cattle production worldwide. Pesticides such as synthetic pyrethroids and organophosphates are routinely used to control horn flies; however, resistance to these chemicals has become a concern in several countries. To further elucidate the molecular mechanisms of resistance in horn fly populations, we sequenced the transcriptomes of ten populations of horn flies from the southern US possessing varying degrees of pesticide resistance levels to pyrethroids, organophosphates, and endosulfans. We employed an Illumina paired end HiSeq approach, followed by *de novo* assembly of the transcriptomes using CLC Genomics Workbench 8.0.1 De Novo Assembler using multiple kmers, and annotation using Blast2GO PRO version 5.2.5. The Gene Ontology biological process term Response to Insecticide was found in all the populations, but at an increased frequency in the populations with higher levels of insecticide resistance. The raw sequence reads are archived in the Sequence Read Archive (SRA) and assembled population transcriptomes in the Transcriptome Shotgun Assembly (TSA) at the National Center for Biotechnology Information (NCBI).

**Specifications Table**

**Table:** 

Subject	Biological sciences
Specific subject area	Omics: Transcriptomics
Type of data	Tables Bar graphs Principal Component Analysis plot Sequence reads Assembled sequences
How the data were acquired	RNA was barcoded and pooled into two lanes then sequenced on Illumina HiSeq 4000 with 2 × 75 standard protocol producing 150bp paired-ends reads at Texas A&M AgriLife Genomics & Bioinformatics Service (College Station, TX, USA).
Data format	Raw Analyzed
Description of data collection	Pools of 10 adult male and 10 adult female horn flies were prepared using 4 replicates from each of the 10 sampled populations with RNA isolated from each replicate using ToTALLY RNA Kit (Thermo Fisher Scientific – Life Technologies, Carlsbad, CA, USA) followed by DNase treatment using TURBO DNA-free Kit (Thermo Fisher Scientific – Life Technologies).
Data source location	- Kerrville Susceptible, Kerrville Resistant & Kerrville Super Resistant Populations from Unites States Department of Agriculture, Agricultural Research Service Knipling-Bushland US Livestock Insects Research Laboratory, Kerrville, TX, United States of America - Georgia Saber Population from University of Georgia Central Branch Experiment Station, Eatonton, GA, United States of America - LSU Hill Farm Fall 2004 Population from Louisiana State University AgCenter Research Station, Homer, LA, United States of America - LSU Red River Fall 1999 Population from Louisiana State University AgCenter Research Station, Bossier City, LA, United States of America - LSU Winnsboro Fall 1997 & Winnsboro Fall 2010 Populations from Louisiana State University AgCenter Research Station, Winnsboro LA, Unites States of America -LSU Rosepine Fall 1998 & Rosepine Spring 2004 Populations from Louisiana State University AgCenter Research Station, Rosepine, LA, United States of America
Data accessibility	Repository name: National Center for Biotechnology Information (NCBI) Direct URL to data: https://www.ncbi.nlm.nih.gov/bioproject/PRJNA30967 Sequence data for this project can be found at National Center for Biotechnology Information (NCBI) under BioProject accession number PRJNA30967. Repository name: Mendeley Data Data identification number: 10.17632/szjkzv8bj6.2 Direct URL to data: https://data.demo-uni.com/datasets/szjkzv8bj6/2 Bendele, Kylie (2023), “Horn fly transcriptome data of 10 populations from the southern United States Supplementary Data”, Demo Uni, V3, doi: 10.17632/szjkzv8bj6.2 Supplementary File 1 - SortMeRNA custom horn fly mitochondrial and ribosomal RNA database created from NCBI sequences to remove most of the overrepresented ribosomal and mitochondrial sequences.
	Supplementary Table 1 - Preassembly statistics of population and replicate read pairs Supplementary Table 2 - De novo transcriptome assembly statistics Supplementary Table 3 - Populations BlastX annotations Supplementary Table 4 - Populations InterProScan annotations Supplementary Table 5 - Populations Gene Ontology (GO) annotations Supplementary Table 6 - Top 10 GO terms from each population Supplementary Table 7 - Populations enzyme code annotations Supplementary Table 8 – Populations BlastN annotations
Related research article	K.G. Bendele, D.M. Bodine, Q. Xu, L.D. Foil, C. Cameron, A. Perez de Leon, A. Farmer, E. Retzel, V. Moore, K.H. Lohmeyer, F.D. Guerrero, The adult horn fly transcriptome and its complement of transcripts encoding cytochrome P450s, glutathione S-transferases, and esterases, Vet. Parasitol. 304: 109699 (2022). https://doi.org/10.1016/j.vetpar.2022.109699

## Value of the data


 

• These are transcriptomes from different populations of parasitic biting flies of livestock with varying levels and mechanisms of phenotypically characterized insecticide resistance.
• Researchers studying insecticide resistance in biting flies will find these assembled transcriptomes valuable due to the expanded gene expression sequence data in the transcripts.
• The datasets produced from these transcriptomes can be used in comparative studies of molecular mechanisms causing insecticide resistance in biting flies.
• The RNASeq data provided in this study can be used with or without a genome for single nucleotide polymorphism (SNP) calling.

## 1 Objective

This dataset is the foundational set for Bendele et al. [1] and their study of molecular mechanisms of horn fly pesticide resistance. Our objective in designing the study and acquiring and analyzing the dataset was to be comprehensive and include all the known horn fly pesticide resistance phenotypes in the fly populations that were sampled. Ten horn fly populations with wide-ranging resistance phenotypes were selected and transcriptomes from each population were synthesized and sequenced. The transcriptomes were analyzed individually and all ten transcriptomes were pooled to produce a single horn fly transcriptome. This dataset adds value to Bendele et al. [1] by providing a foundation of 10 transcriptomes for more targeted molecular studies of a wide range of genes in pesticide resistant and susceptible horn flies, including genes with roles in pesticide metabolism and detoxification.

## 2 Data Description

### 2.1 Raw data

**Fig. 1:**
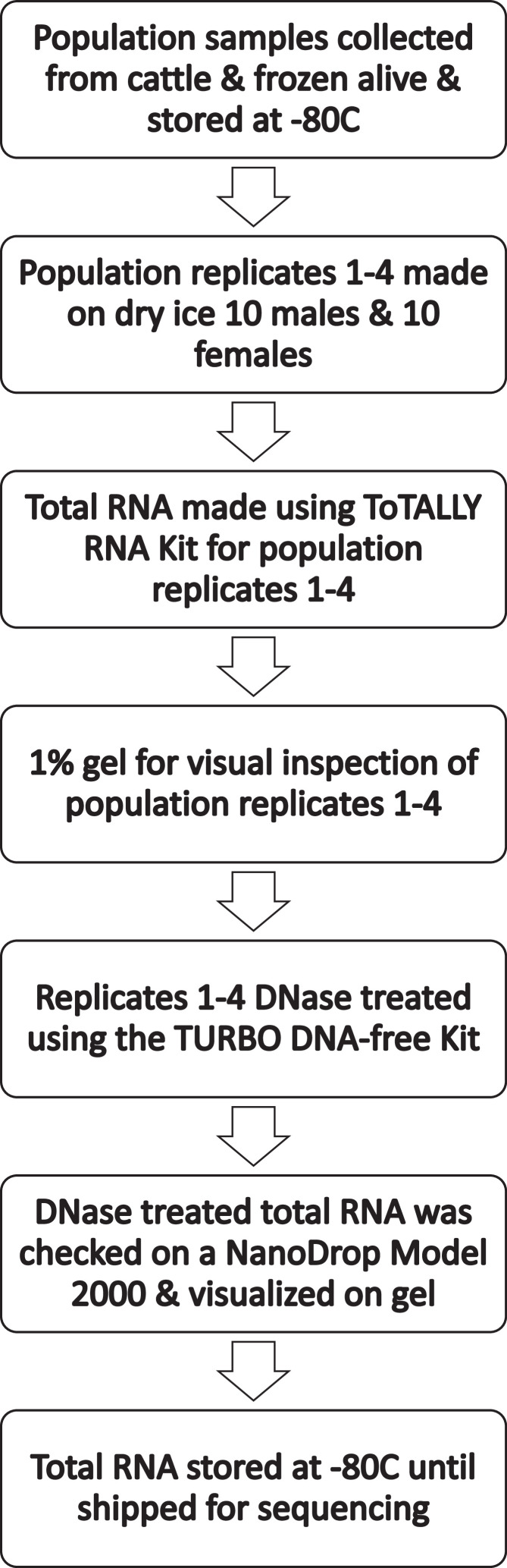
RNA samples experimental design flowchart.

**Fig. 2:**
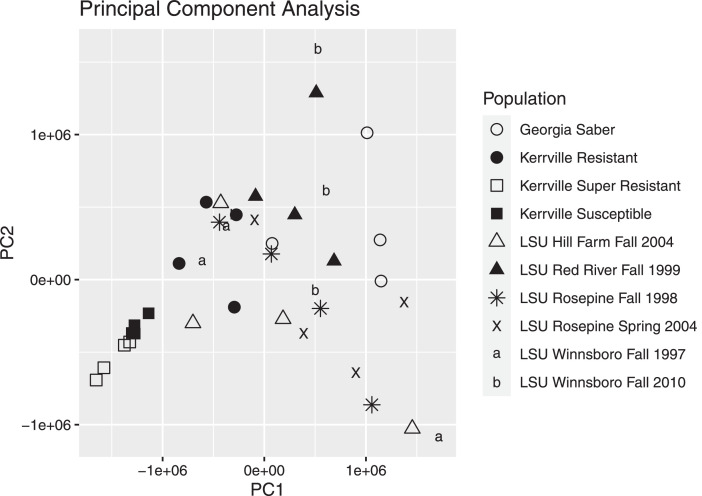
Principal Component Analysis plot of population replicates.

Population samples were collected from the backs of cattle using the aerial hand net method and were frozen while alive and stored at −80 °C until the population samples were sorted to make the RNA population samples. Horn fly samples were sorted by sex on dry ice and 10 individual male horn flies, and 10 individual female horn flies pooled and placed into tubes pre-chilled on dry ice. A total of 4 replicates from each population were created, with the total fly masses shown in Table 2. To increase the statistical power of the data sets, 4 replicates were prepared for each population. RNA was isolated from each replicate using the ToTALLY RNA Kit (Thermo Fisher Scientific – Life Technologies, Carlsbad, CA, USA) following the manufacturer's recommendation with each replicate sample being ground in liquid nitrogen using a baked mortar and pestle. Total RNA samples were run on 1% agarose gel for visual inspection then DNase treated using the TURBO DNA-free Kit (Thermo Fisher Scientific – Life Technologies) according to the manufacturer's recommendations. The DNase treated total RNA was checked on a NanoDrop Model 2000 with the 260/280 reading listed in Table 2 and checked on a 1% agarose gel for a final visual inspection before storage at −80 °C with Fig. 1 showing an overview of the process. RNA was barcoded and pooled, then sequenced on 2 lanes of Illumina HiSeq 4000 with a 75 × 75 standard protocol producing 150bp paired-ends reads. The raw reads were deposited at NCBI under BioProject PRJNA30967, BioSample accession numbers SAMN11539754- SAMN11539763, SRA accession numbers SRR9016890- SRR9016899 and as SRA study SRP000249 as listed in Table 3. A Principal Component Analysis was conducted on individual replicate read counts aligned to the Kerrville Susceptible transcriptomes using Bowtie2 and R using R Studio 2022.12.0+353 using the tidyverse package (Fig. 2).

**Table 1:** Collection locations and resistance phenotype of horn fly populations.

		Resistance status		
Population name	Location	Class & Phenotype	Mechanisms (If known)	Reference
Kerrville Susceptible	Kerrville, TX, USA	Pyrethroid Susceptible	-	[2]
		Organophosphate Susceptible	-	
Kerrville Resistant	Kerrville, TX, USA	Pyrethroid Moderate	Target site + Metabolic	[2]
		Organophosphate Susceptible	-	
Kerrville Super Resistant	Kerrville, TX, USA	Pyrethroid Very high	Target site + Metabolic	[2]
		Organophosphate Susceptible	-	
Georgia Saber	Eatonton, GA, USA	Pyrethroid High	Target site + Metabolic	[3,4]
		Organophosphate Unknown	Unknown	
LSU Hill Farm Fall 2004	Homer, LA, USA	Pyrethroid High	Target site	[5]
		Organophosphate High	Unknown	
LSU Red River Fall 1999	Red River, LA, USA	Pyrethroid High	Target site	[6]
		Organophosphate Low	Unknown	
LSU Winnsboro Fall 1997	Winnsboro, LA, USA	Pyrethroid Very high	Unknown	[7]
		Organophosphate Moderate	Unknown	
LSU Winnsboro Fall 2010	Winnsboro, LA, USA	Pyrethroid Moderate	Target site	[8]
		Organophosphate High	Unknown	
		Endosulfan High	Target site	
LSU Rosepine Fall 1998	Rosepine, LA, USA	Pyrethroid Low	Unknown	[5]
		Organophosphate Moderate	Unknown	
LSU Rosepine Spring 2004	Rosepine, LA, USA	Pyrethroid Low	Unknown	[5]
		Organophosphate Moderate	Unknown

**Table 2:** Horn fly population replicates RNA isolation data.

Population Replicate	Total Fly Mass (mg)	NanoDrop 260/280 Reading
Kerrville Susceptible 1	120	2.25
Kerrville Susceptible 2	130	2.26
Kerrville Susceptible 3	110	2.24
Kerrville Susceptible 4	140	2.26
Kerrville Resistant 1	100	2.27
Kerrville Resistant 2	100	2.27
Kerrville Resistant 3	100	2.24
Kerrville Resistant 4	90	2.23
Kerrville Super Resistant 1	120	2.22
Kerrville Super Resistant 2	120	2.21
Kerrville Super Resistant 3	120	2.17
Kerrville Super Resistant 4	120	2.25
Georgia Saber 1	110	2.26
Georgia Saber 2	97	2.23
Georgia Saber 3	100	2.23
Georgia Saber 4	100	2.23
LSU Hill Farm Fall 2004 1	80	2.28
LSU Hill Farm Fall 2004 2	90	2.27
LSU Hill Farm Fall 2004 3	90	2.27
LSU Hill Farm Fall 2004 4	80	2.27
LSU Red River Fall 1999 1	110	2.25
LSU Red River Fall 1999 2	110	2.27
LSU Red River Fall 1999 3	110	2.23
LSU Red River Fall 1999 4	110	2.26
LSU Winnsboro Fall 1997 1	90	2.23
LSU Winnsboro Fall 1997 2	100	2.25
LSU Winnsboro Fall 1997 3	80	2.23
LSU Winnsboro Fall 1997 4	100	2.23
LSU Winnsboro Fall 2010 1	110	2.26
LSU Winnsboro Fall 2010 2	100	2.26
LSU Winnsboro Fall 2010 3	70	2.25
LSU Winnsboro Fall 2010 4	90	2.26
LSU Rosepine Fall 1998 1	100	2.25
LSU Rosepine Fall 1998 2	90	2.26
LSU Rosepine Fall 1998 3	100	2.26
LSU Rosepine Fall 1998 4	110	2.26
LSU Rosepine Spring 2004 1	130	2.26
LSU Rosepine Spring 2004 2	120	2.25
LSU Rosepine Spring 2004 3	110	2.25
LSU Rosepine Spring 2004 4	120	2.24

**Table 3:** Horn fly populations NCBI accession numbers and assembly statistics.

	BioSample	Sequence Read	Transcriptome	Total Read	Transcript
Population	Accession No.	Accession No.	Accession No.	Pairs -Trimmed	No.
Kerrville Susceptible	SAMN11539761	SRR9016894	GHLQ01000000	100,061,801	48,505
Kerrville Resistant	SAMN11539757	SRR9016898	GHWU01000000	77,757,708	44,547
Kerrville Super Resistant	SAMN11539760	SRR9016895	GHXD01000000	97,656,382	48,558
Georgia Saber	SAMN11539754	SRR9016897	GHLV01000000	89,493,214	67,443
LSU Hill Farm Fall 2004	SAMN11539755	SRR9016896	GHMY01000000	83,149,678	62,303
LSU Red River Fall 1999	SAMN11539756	SRR9016899	GHNS01000000	89,522,025	71,783
LSU Winnsboro Fall 1997	SAMN11539763	SRR9016890	GHYQ01000000	87,835,135	69,714
LSU Winnsboro Fall 2010	SAMN11539762	SRR9016891	GHXP01000000	86,391,817	57,912
LSU Rosepine Fall 1998	SAMN11539758	SRR9016893	GIBC01000000	88,702,755	71,883
LSU Rosepine Spring 2004	SAMN11539759	SRR9016892	GIDC01000000	77,226,036	57,045

### 2.2 Assembled transcriptomes

**Fig. 3:**
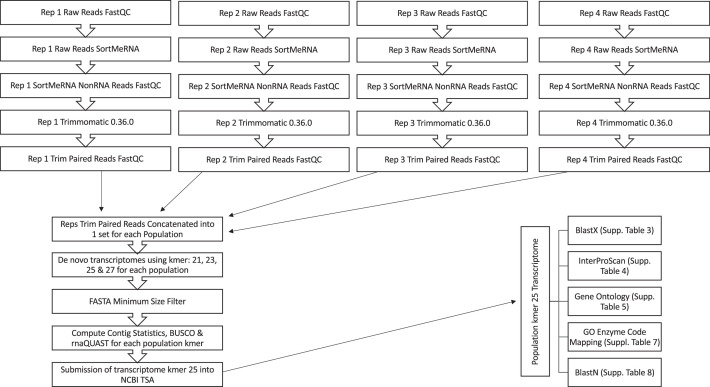
Annotation experimental design flowchart.

Each population had four replicates sequenced with each replicate treated individually for all preassembly bioinformatic steps and then pooled for the transcriptome assembly with Fig. 3 showing an overview of the analysis steps. The highest quality *de novo* CLC Genomic Workbench 8.0.1 transcriptomes were produced using kmer 25. The assembled transcriptomes from each population were submitted to NCBI Transcriptome Shotgun Assembly (TSA) database with accession numbers GHLQ01000000 (Kerrville Susceptible), GHWU01000000 (Kerrville Resistant), GHXD01000000 (Kerrville Super Resistant), GHLV01000000 (Georgia Saber), GHMY01000000 (LSU Hill Farm 2004), GHNS01000000 (LSU Red River Fall 1999), GHYQ01000000 (LSU Winnsboro Fall 1997), GHXP01000000 (LSU Winnsboro Fall 2010), GIBC01000000 (LSU Rosepine Fall 1998) and GIDC01000000 (LSU Rosepine Spring 2004).

## 3 Experimental Design, Materials and Methods

### 3.1 Sequencing

Ten horn fly populations with varying levels of phenotypically diagnosed insecticide resistance (Table 1) were sequenced in a research study on horn fly insecticide resistance mechanisms [1]. Pools of 10 adult male and 10 adult female horn flies were prepared, using 4 replicates of each population. RNA was isolated from each replicate using TRIzol Reagent (Thermo Fisher Scientific) followed by DNase treatment using the RNeasy kit (Qiagen) following manufacturers’ recommended protocols. The RNA was sequenced at Texas A&M AgriLife Genomics and Bioinformatics Service (College Station, TX, USA), barcoding all samples and pooling into two lanes of the Illumina HiSeq 4000 with a 2 × 75 standard protocol producing 150bp paired end reads.

### 3.2 Transcriptome assembly

Raw read files were checked for quality using FastQC version 0.11.5, which showed a large amount of contamination of ribosomal and mitochondrial RNA as overrepresented sequences. SortMeRNA version 2.1 [9] was used with a custom horn fly mitochondrion and ribosomal database created from NCBI accession numbers KM669714, DQ029097, NC_007102, EU375511, EU375513, EU375514, HQ844235, DQ437515, EF560184, U60809, JQ246755, JQ246651, EU179518, EU013947, EU013946, FJ025436, KJ470673, KJ470672, KJ470671 and KJ470670 in Supplementary File 1 at Mendeley Data [10]. The remaining reads were uploaded into CyVerse Discovery Environment (DE) [11,12] using Cyberduck version 6.0.4 then rechecked using FastQC 0.11.5 (multi-file) app. The CyVerse DE Trimmomatic 0.36.0 [13] app was used with Trimmomatic TruSeq2-PE adapter file and all default parameters with the addition of a 14bp read head crop length. The resulting Trimmomatic output files were checked for the final time with FastQC 0.11.5 (multi-file) app. Preassembly read statistics for each population and replicate can be found in Supplementary Table 1 at Mendeley Data [10]. The population replicates read files were sequenced with each of the replicates treated individually for all preassembly bioinformatic steps and then concatenated into a pair of read files (R1 & R2) for each population using Concatenate Multiple Files DE app available in CyVerse DE for the transcriptome assembly.

*De novo* transcriptomes were assembled for each population using the CLC Genomics Workbench 8.0.1 (Qiagen) De Novo Assembler using word size/kmer of 21, 23, 25, and 27 with bubble size of 75, minimum contig length of 200, and default mapping options (mismatch cost of 2, insertion cost of 3, length fraction of 0.5 and similarity fraction of 0.8). The *de novo* transcriptome assemblies were filtered using FASTA Minimum Size Filter app in CyVerse DE to remove any sequences of less than 200bp. CyVerse DE apps Compute Contig Statistics, BUSCO-v3.0 [14] with diperta_odb9 lineage, and rnaQUAST_1.2.0 (*de novo* based) [15] were used with default parameters to assess the quality of the different word size/kmer transcriptome assemblies and results tabulated in Supplementary Table 2 at Mendeley Data [10]. The word size/kmer 25 transcriptomes were determined as the highest quality and submitted to NCBI as part of the Transcriptome Shotgun Assembly (TSA) database.

### 3.3 Transcriptome annotations

**Fig. 4:**
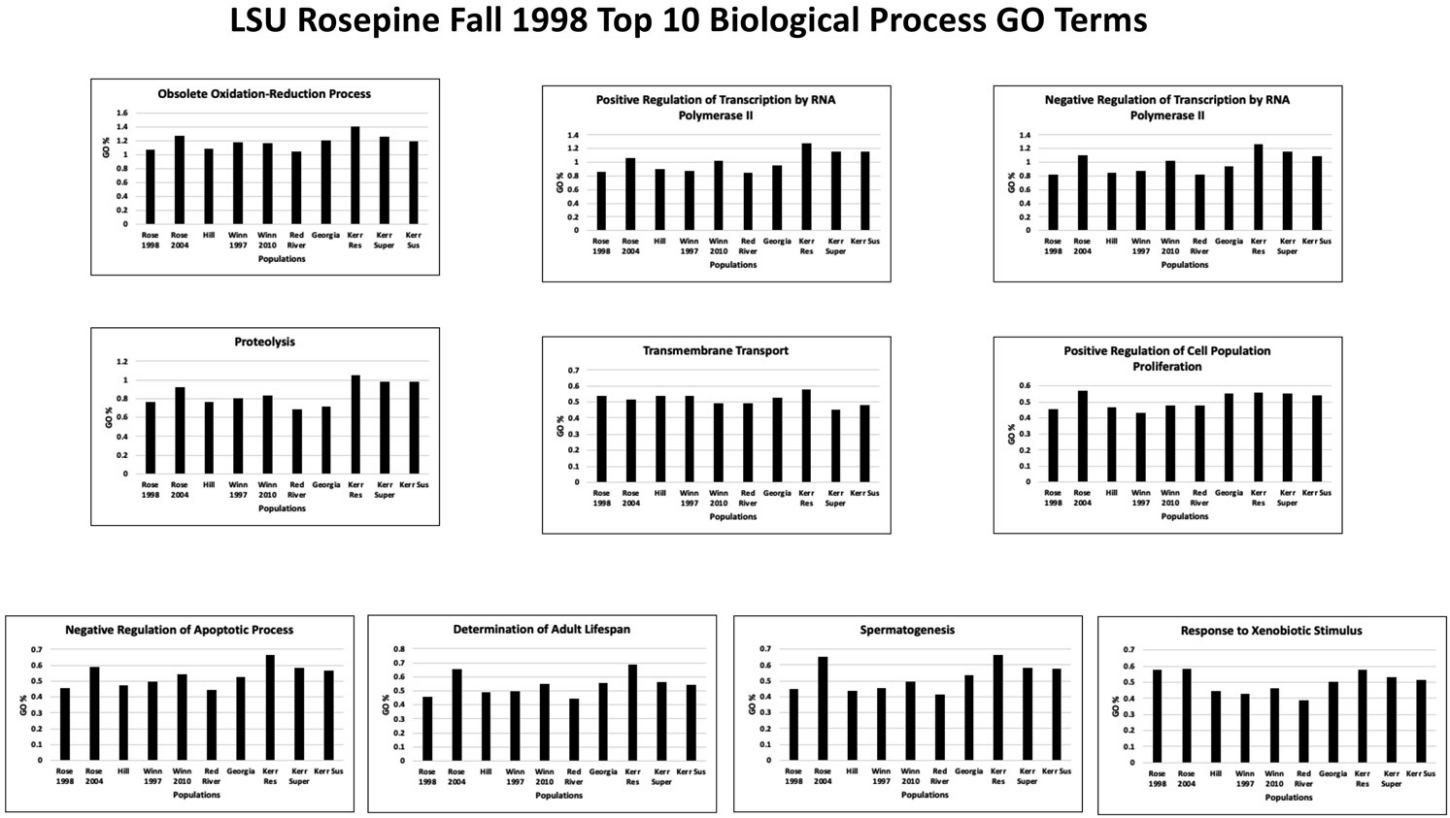
The top ten Biological Process Gene Ontology terms of the LSU Rosepine Fall 1998 population. Each top ten Rosepine Fall 1998 GO term is plotted, showing the GO term's % of total GO term count in each of the ten populations.

**Fig. 5:**
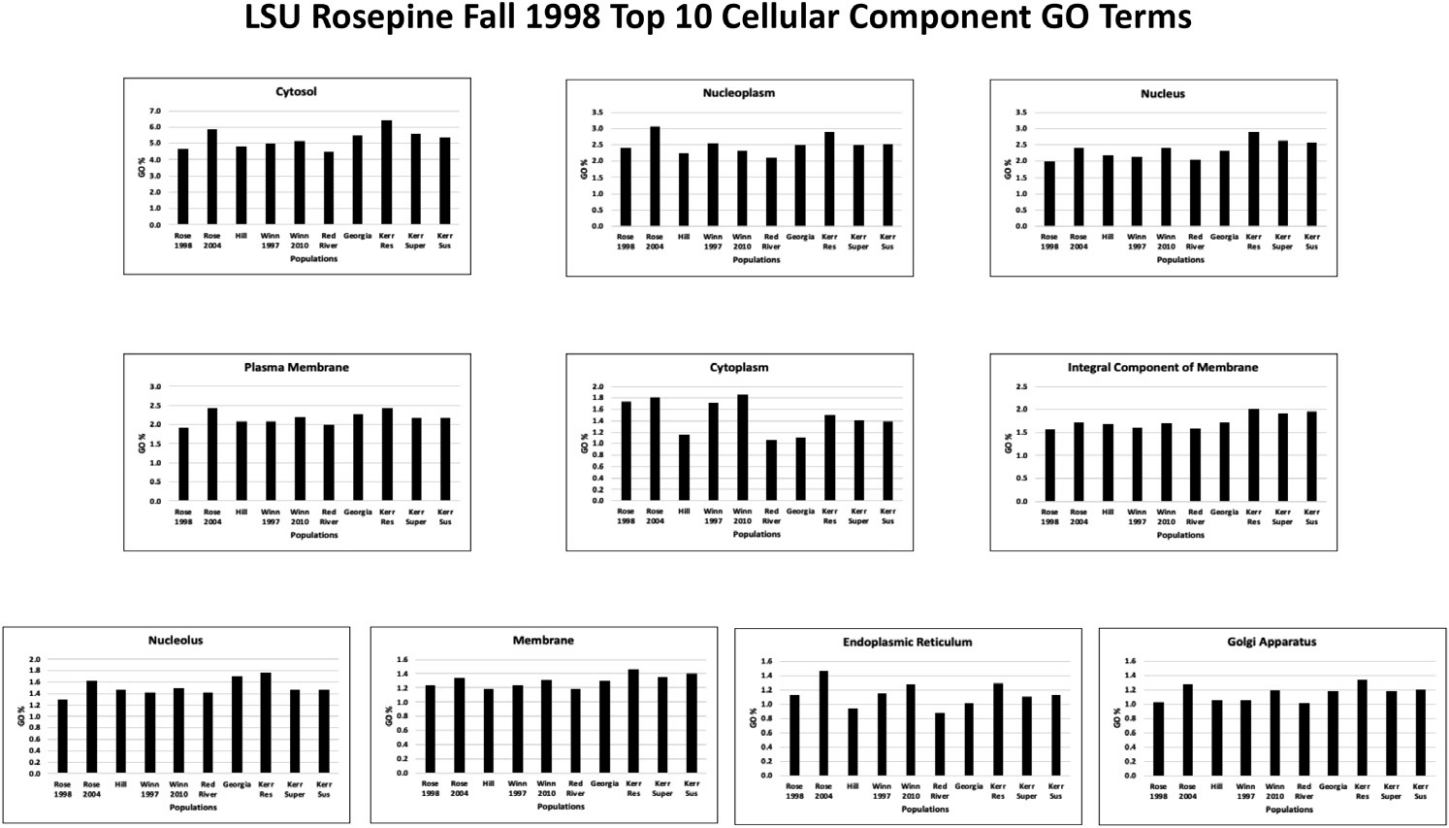
The top ten Cellular Component Gene Ontology terms of the LSU Rosepine Fall 1998 population. Each top ten Rosepine Fall 1998 GO term is plotted, showing the GO term's % of total GO term count in each of the ten populations.

**Fig. 6:**
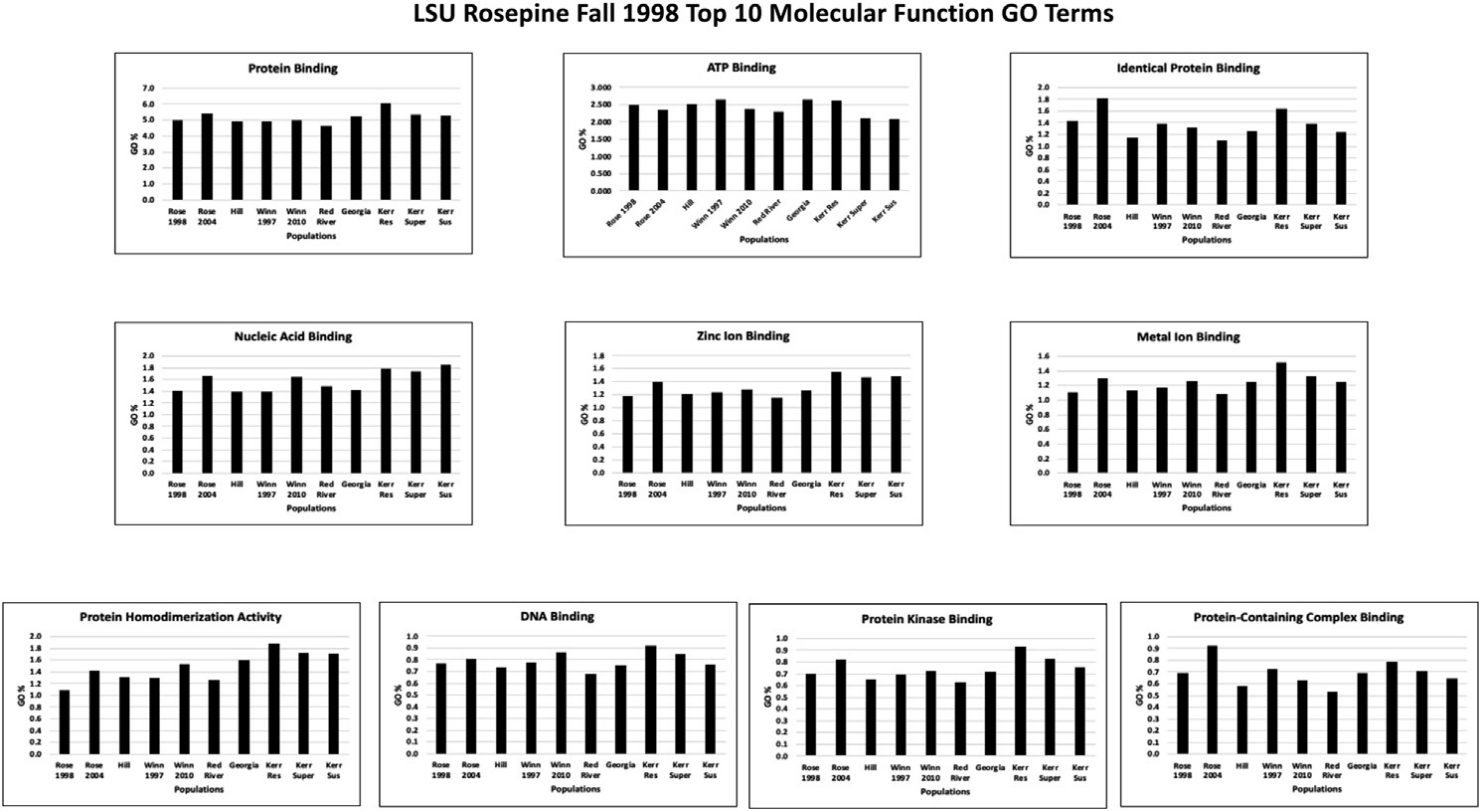
The top ten Molecular Function Gene Ontology terms of the LSU Rosepine Fall 1998 population. Each top ten Rosepine Fall 1998 GO term is plotted, showing the GO term's % of total GO term count in each of the ten populations.

Each of the population TSA transcriptomes was annotated using BLAST2GO PRO version 5.2.5 [16], [17], [18], [19] using CloudBlast to perform BlastX against the UniProtKB/Swiss-Prot database with an e-value of 1.0E-25 and default parameters (Supplementary Table 3 at Mendeley Data [10]). InterProScan searches were performed using CloudIPS for all families, domains, sites, repeats, structural domains, and other sequence features (Supplementary Table 4 at Mendeley Data [10]). Gene Ontology (GO) mapping was done using database version 2019.04 followed by GO annotation using default parameters except Blast E-value hit filter 1.0E-25 to match the E-value used for BlastX searches noted earlier (Supplementary Table 3 at Mendeley Data [10]). The InterProScan GO annotations are provided in Supplementary Table 5, with the top ten Biological Process, Cellular Component, and Molecular Function GO terms from each population shown in Supplementary Table 6 at Mendeley Data [10]. The top ten Biological Process, Cellular Component, and Molecular Function GO terms from these 10 different populations based on the LSU Rosepine Fall 1998 population are shown in Fig. 4, Fig. 5, Fig. 6, respectively. GO Enzyme Code Mapping was done using default parameters and can be found in Supplementary Table 7 at Mendeley Data [10]. Each transcriptome was annotated using blast+/2.13.0 with default BlastN parameters against the NCBI Nucleotide Sequence Database (NT 3-14-2023) then further filtered by an e-value 1.0E-25 and results can be found in Supplementary 8 at Mendeley Data [10].

## Ethics Statements

No experiments were conducted on animals for this manuscript.

## CRediT authorship contribution statement

**Kylie G. Bendele:** Validation, Formal analysis, Investigation, Data curation, Writing – original draft, Visualization. **Felix D. Guerrero:** Conceptualization, Methodology, Investigation, Resources, Data curation, Writing – review & editing, Visualization, Supervision, Project administration, Funding acquisition. **Kimberly H. Lohmeyer:** Writing – review & editing, Supervision, Funding acquisition. **Lane D. Foil:** Conceptualization, Resources, Writing – review & editing. **Richard P. Metz:** Methodology, Software, Writing – review & editing. **Charles D. Johnson:** Conceptualization, Methodology, Writing – review & editing.

## Declaration of Competing Interest

The authors declare that they have no known competing financial interests or personal relationships that could have appeared to influence the work reported in this paper.

## Data Availability

Supplementary Table 3 (Original data) (Mendeley Data). Supplementary Table 8 (Original data) (Mendeley Data). TSA Georgia Saber (Original data) (NCBI). TSA LSU Red River Fall 1999 (Original data) (NCBI). TSA LSU Rosepine Fall 1998 (Original data) (NCBI). TSA LSU Rosepine Spring 2004 (Original data) (NCBI). BioSample Kerrville Resistant (Original data) (NCBI). SRA Kerrville Resistant (Original data) (NCBI). BioSample Kerrville Super Resistan (Original data) (NCBI). SRA Kerrville Super Resistan (Original data) (NCBI). BioSample Georgia Saber (Original data) (NCBI). SRA Georgia Saber (Original data) (NCBI). BioSample LSU Hill Farm 2004 (Original data) (NCBI). SRA LSU Hill Farm 2004 (Original data) (NCBI). BioProject (Original data) (NCBI). BioSample Kerrville Susceptible (Original data) (NCBI). SRA Kerrville Susceptible (Original data) (NCBI). BioSample LSU Rosepine Spring 2004 (Original data) (NCBI). SRA LSU Rosepine Spring 2004 (Original data) (NCBI). TSA Kerrville Super Resistant (Original data) (NCBI). TSA LSU Hill Farm 2004 (Original data) (NCBI). TSA LSU Winnsborro Fall 1997 (Original data) (NCBI). TSA LSU Winnsborro Fall 2010 (Original data) (NCBI). TSA Kerrville Susceptible (Original data) (NCBI). TSA Kerrville Resistant (Original data) (NCBI). BioSample LSU Red River Fall 1999 (Original data) (NCBI). SRA LSU Red River Fall 1999 (Original data) (NCBI). BioSample LSU Winnsborro Fall 1997 (Original data) (NCBI). SRA LSU Winnsborro Fall 1997 (Original data) (NCBI). SRA LSU Winnsborro Fall 2010 (Original data) (NCBI). BioSample LSU Winnsborro Fall 2010 (Original data) (NCBI). BioSample LSU Rosepine Fall 1998 (Original data) (NCBI). SRA LSU Rosepine Fall 1998 (Original data) (NCBI).
